# Epidemiology of laboratory-confirmed mumps infections in South Africa, 2012–2017: a cross-sectional study

**DOI:** 10.1186/s12889-020-08835-x

**Published:** 2020-05-12

**Authors:** Mpho Lerato Sikhosana, Lazarus Kuonza, Nkengafac Villyen Motaze

**Affiliations:** 1 South African Field Epidemiology Training Programme, National Institute for Communicable Diseases, Division of the National Health Laboratory Service, Johannesburg, South Africa; 2 Division of Public Health Surveillance and Response, National Institute for Communicable Diseases, Division of the National Health Laboratory Service, Johannesburg, South Africa; 3 School of Health Systems and Public Health, University of Pretoria, Pretoria, South Africa; 4 Centre for Vaccines and Immunology, National Institute for Communicable Diseases, a division of the National Health Laboratory Service, Johannesburg, South Africa; 5 Department of Global Health, Faculty of Medicine and Health Sciences, Stellenbosch University, Stellenbosch, South Africa

**Keywords:** Mumps, Epidemiology, Public health sector, Private health sector, Acute infections, Immunity

## Abstract

**Background:**

Data on the burden of mumps in South Africa are limited and the epidemiology of mumps in this setting is not well understood. We present an analysis of mumps data in South Africa from 2012 to 2017.

**Methods:**

This cross-sectional study included secondary data on laboratory-confirmed mumps infections from 2012 to 2017, archived at the South African National Health Laboratory Services’ data repository as well as from four private laboratories. Mumps-specific immunoglobulin M (IgM) and/or viral nucleic acid positive results represented acute infections. We used age-specific mid-year population estimates for each study year as denominators when calculating annual cumulative incidence. Seasonality was based on the season that showed a peak in infections.

**Results:**

Out of 48,580 records obtained from the public and private sectors, 46,713 (96.2%) were from the private sector. Over the study period, there were 7494 acute infections, 7085 (94.5%) of which were recorded in the private sector. Of these 7494 infections, 3924 (52.4%) occurred in males. The proportion of samples tested that were IgM positive was 18.6% (1058/5682) in 2012, 15% (1016/6790) in 2013, 15.8% (1280/8093) in 2014, 15.5% (1384/8944) in 2015, 13.1% (1260/9629) in 2016 and 15.8% (1496/9442) in 2017. The cumulative incidence rate per 100,000 was highest in children between one and 9 years throughout the study period. The cumulative incidence of infections was highest in the Western Cape, Gauteng and the Northern Cape. Infections peaked in June and November.

**Conclusion:**

Laboratory-confirmed mumps infections predominantly occurred in spring, affecting children below 10 years of age and individuals who were male. There were fewer tests performed in the public sector compared to the private sector. Since only laboratory data was analysed our results represent and underestimate of disease burden. Further studies that include clinical data are required to provide better estimates of disease burden in South Africa.

## Background

Mumps is usually a childhood illness that mostly affects children aged 5–9 years although adolescents and adults can be infected [1]. In the absence of a mumps-containing vaccine (MuCV), the annual incidence of mumps was estimated to be between 100 and 1000 cases/100000 population [1]. By the end of 2018, 122 countries worldwide had introduced the vaccine in their respective national immunization programmes, with the annual reported number of cases being 499,512 worldwide [2, 3]. However, there has been reports of mumps outbreaks in previously adequately vaccinated populations recently, indicating possible waning immunity [4–10].

Mumps is not a notifiable disease in South Africa [11]. This contributes to the sparse epidemiological data about the disease and the baseline incidence of mumps infections not being known. Between 1999 and 2018, only two Southern African countries (Eswatini and Zambia) frequently reported mumps cases, while South Africa only reported 24 cases in 2002 [12].

Important policy and programmatic considerations regarding the introduction of a MuCV into a country’s immunization programme include the burden of mumps disease, the efficiency of the country’s national immunization programme, the socioeconomic impact of the vaccine introduction, and the ability of the country to achieve and maintain a coverage > 80% for the measles- and rubella-containing vaccines [13]. A vaccine coverage > 80% for the measles- and rubella-containing vaccines demonstrates a country’s ability to achieve a similar or higher coverage for the MuCV. A suboptimal coverage would result in an epidemiological shift of disease, leading to a higher incidence of mumps infections in the older age-groups. An increase in age has been associated with more severe disease in many childhood diseases, and in mumps infections, this increased risk occurs more commonly in males compared to females [10, 14] The World Health Organization (WHO) also recommends that should a MuCV be introduced by a country, it should be as two doses given at 12–18 months then from 2 up to 6 years in the form of the trivalent measles-mumps-rubella (MMR) vaccine. Mumps should also be included in the country’s list of notifiable diseases that would be under surveillance. By 2017, only four countries in the African region (Seychelles, Mauritius, Cabo Verde and Algeria) had introduced the MuCV in their respective national vaccination program, while in South Africa, MuCV was only available in the private health sector as MMR and was not part of the Extended Programme of Immunizations (EPI) that provides vaccines to children in the public health sector [15–17].

We therefore aimed to describe the epidemiology of laboratory-confirmed mumps infections in South Africa between January 2012 to December 2017, with the objectives of estimating the cumulative incidence of laboratory-confirmed infections as well as determining whether the infections had periodic fluctuations.

## Methods

### Study design and setting

This was a cross-sectional study using laboratory data from both private and public health sectors, from January 2012 to December 2017. The study period was chosen because mumps data was recorded more consistent in both health sectors during this period. Public sector data were obtained from the data repository of the National Health Laboratory Service (NHLS). The NHLS is the largest diagnostic pathology service provider in South Africa and provides laboratory services to ≥80% of the population through a network of over 260 laboratories throughout the country [18]. Private sector data were obtained from four private laboratories (Ampath, Lancet, PathCare and Vermaak & Partners). Data included patient’s demographic information as well as test results.

### Operational definitions

Positive mumps-specific immunoglobulin M (IgM) and/or viral nucleic acid (NA) results represented acute infections. Results positive only for mumps-specific immunoglobulin G (IgG) represented previous exposure to mumps. Age-specific cumulative incidence rates were calculated using the number of acute infections per year as the numerator and the age-specific mid-year population estimates as denominators [19–24]. Mid-year population estimates for 2012 were not available, therefore the average of estimates for 2011 and 2013 were used to calculate the 2012 age-specific estimates. A seasonal pattern was determined by the months that showed a peak in the number of infections.

### Participants, sample size and sampling

All samples tested for mumps at the NHLS and the four private laboratories during the study period were included.

### Data management and analysis

Stata statistical software version 15 (StataCorp. 2017. Stata Statistical Software: Release 15. College Station, TX: StataCorp LLC) was used for data cleaning and analysis.

### Ethical considerations

Ethics approval for conducting this study was obtained from the Faculty of Health Sciences Research Ethics Committee of the University of Pretoria (ref. 539/2017). Institutional clearance was also obtained from the NHLS Academic Affairs, Research and Quality Assurance as well as the relevant ethics committees of the respective private laboratories.

## Results

**Fig. 1:**
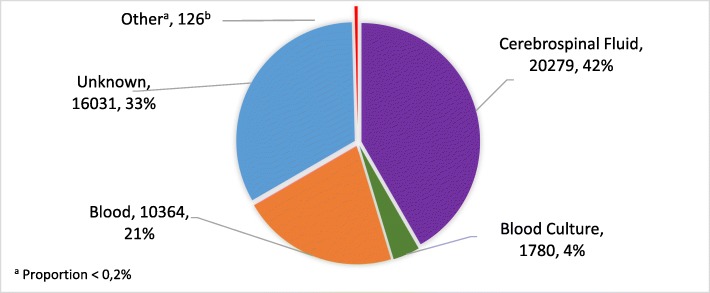
Types of specimens submitted for mumps testing in the public and private sectors, 2012–2017. ^b^Other: swabs, stool, sears, saliva/sputum, nasopharyngeal aspirates, bone marrow, amniotic fluid

A total of 48,580 records were used in the analysis. Participant characteristics are summarized in Table 1. Of these records, 46,713 (96.2%) were from the private sector. There were 186 (0.4%) records with missing information on age, 143 (0.3%) on gender, 15,993 (32.9%) on sample type and 15,175 (31.2%) on province. There were 26,640 (54.8%) records from samples collected from females. There were 10,279 samples from children < 9 years, of which 9583 (93.2%) were from the private sector. Types of specimens submitted to both health sectors over the study period are shown in Fig. 1. There were 20,279 (41.7%) cerebrospinal fluid samples, and 12,144 (25.0%) blood samples (this includes samples labelled “blood” and “blood culture”) (Fig. 1). The highest number of samples submitted overall was from Gauteng Province 16,959 (50.8%), while the lowest number of samples was from the Eastern Cape, Northern Cape and Free State Provinces (343 (1%), 345 (1%) and 349 (1%) respectively).

**Table 1:** Characteristics of samples submitted for mumps testing in public and private sectors, 2012–2017, (*n* = 48,580)

Variable	Public Sector, n (%)	Private Sector, n (%)	Total n (%)
**Gender**			
Female	1046 [4]	25,594 (96)	**26,640 (100)**
Male	788 (4)	21,009 (96)	**21,797 (100)**
**Age**			
< 1	139 (9)	1423 (91)	**1562 (100)**
1–4	317 (7)	4080 (93)	**4397 (100)**
5–9	240 (6)	4080 (94)	**4320 (100)**
10–19	263 (4)	6061 (96)	**6324 (100)**
20–29	299 (4)	7184 (96)	**7483 (100)**
30–39	214 (2)	9866 (98)	**10,080 (100)**
> 40	227 (2)	14,001 (98)	**14,228 (100)**
Unknown	168 (90)	18 (10)	**186 (100)**
**Province**			
EC	71 (21)	272 (79)	**343 (100)**
FS	63 (18)	286 (82)	**349 (100)**
GP	933 (6)	16,026 (94)	**16,959 (100)**
KZN	193 (2)	8116 (98)	**8309 (100)**
LP	51 (6)	822 (94)	**873 (100)**
MP	114 (8)	1245 (92)	**1359 (100)**
NW	67 (9)	663 (91)	**730 (100)**
NC	60 (17)	285 (83)	**345 (100)**
WC	315 (8)	3823 (92)	**4138 (100)**

*EC* Eastern Cape; *FS* Free State; *GP* Gauteng; *KZN* KwaZulu Natal; *LP* Limpopo; *MP* Mpumalanga; *NW* North West; *NC* Northern Cape; *WC* Western Cape

**Fig. 2:**
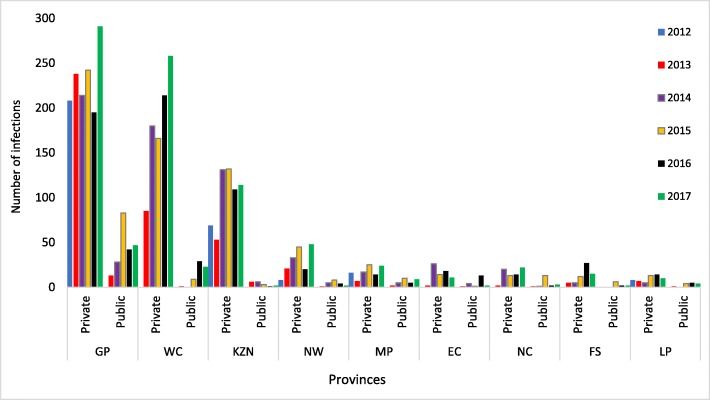
Absolute number of laboratory-confirmed acute mumps infections recorded in the public and private sectors by province, 2012–2017

**Fig. 3:**
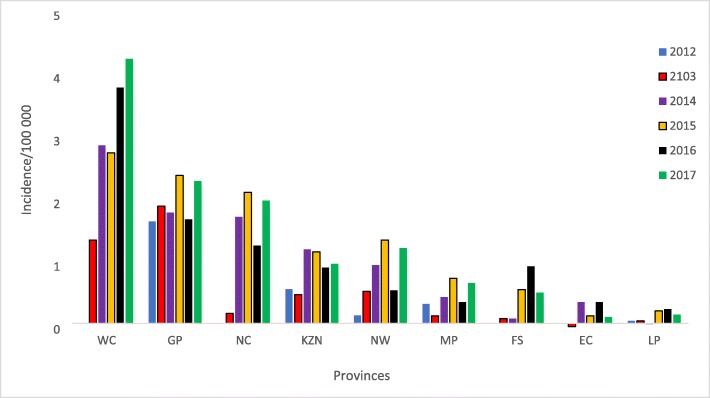
Incidence per 100,000 population of laboratory-confirmed mumps infections in the public and private sectors by province, 2012–2017

**Fig. 4:**
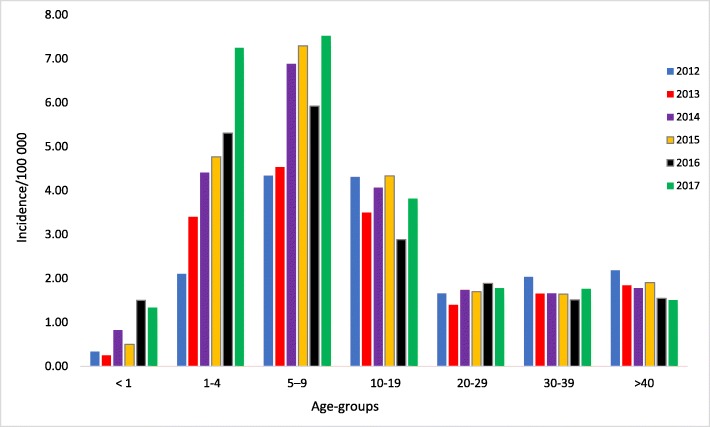
Incidence per 100,000 population of laboratory-confirmed mumps infections in the public and private sectors by age-group, 2012–2017

**Fig. 5:**
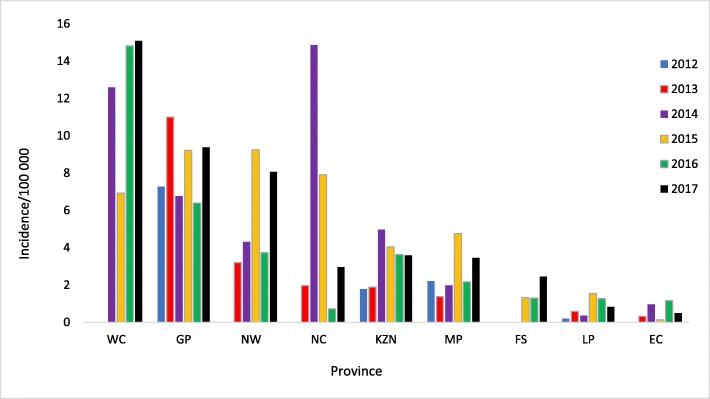
Incidence per 100,000 of laboratory-confirmed acute infections recorded in the public and private sectors by province, 1–4 year age-group, 2012–2017

**Fig. 6:**
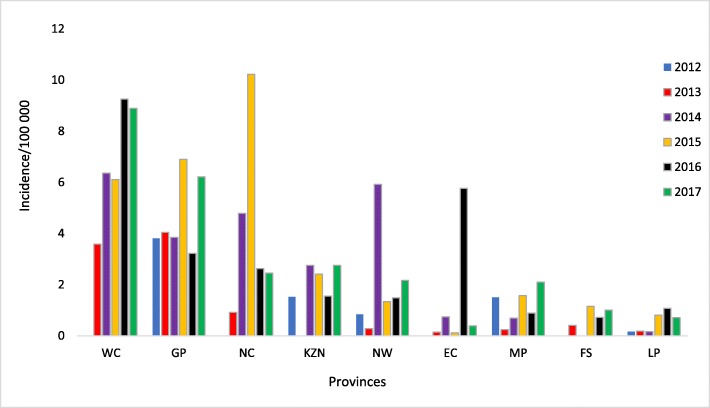
Incidence per 100,000 of laboratory-confirmed acute infections recorded in the public and private sectors by province, 5–9 year age-group, 2012–2017

**Fig. 7:**
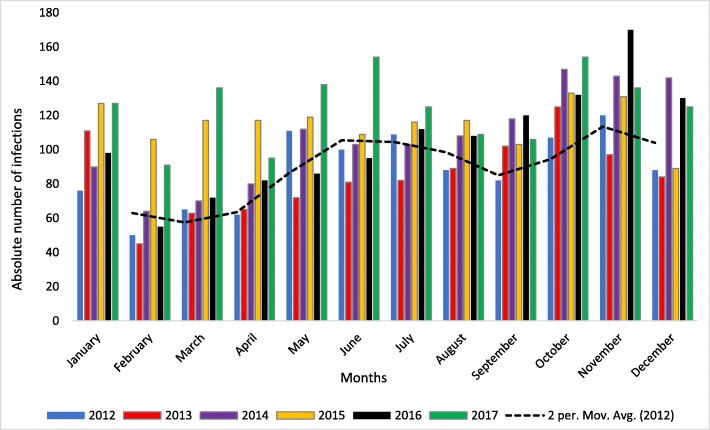
Absolute numbers of laboratory-confirmed acute infections recorded in the public and private sectors by province by month, 2012–2017

Overall, there were 7494 infections recorded during the study period, 7085 (94.5%) of which were in the private sector (Fig. 2). Most of these infections were recorded in 2017 (1496/7494; 20%), while the least number of infections occurred in 2013 (1016/7494; 13.6%). Most (3061/3198; 95.7%) of the infections were diagnosed from blood samples. Except for 2013, there was consistently more infections amongst males (Table 2). The highest absolute numbers of acute infections were recorded in the Gauteng (1601), Western Cape (965) and KwaZulu Natal (626) provinces overall (Fig. 2), however the highest cumulative incidence per 100,000 of infections occurred in Western Cape, Gauteng and Northern Cape, in that order (Fig. 3). On average, the cumulative incidence was highest in 2017 (1,43 cases/100000 population) and lowest in 2013 (0,60 cases/100000 population). The cumulative incidence was high amongst children in the 1–4 and 5–9 year age groups (Fig. 4). When the cumulative incidences of mumps infections in these two most affected age-groups were plotted according to geographic distribution, the highest incidence was in the Western Cape (Figs. 5 and 6). When the absolute numbers of infections were plotted by month, two peaks were observed in June and November throughout the study period (Fig. 7). The province with the highest proportion of cases with evidence of previous exposure to mumps exposure throughout the study period was Gauteng (Table 3).

**Table 2:** Absolute number and percentages of infections reported in public and private sectors by gender, 2012–2017

Sex	Female n (%)	Male n (%)	Total n (%)
**2012**	497 (47,02)	560 (52,98)	**1057 (100)**
**2013**	517 (51,14)	494 (48,86)	**1011 (100)**
**2014**	598 (46,79)	680 (53,21)	**1278 (100)**
**2015**	635 (46,01)	745 (53,99)	**1380 (100)**
**2016**	599 (47,65)	658 (52,35)	**1257 (100)**
**2017**	703 (47,18)	787 (52,82)	**1490 (100)**
**Total**	**3549 (47,49)**	**3924 (52,51**	**7473 (100)**

**Table 3:** Proportion of samples submitted for mumps testing that showed previous exposure in the public and private health sectors, 2012–2017

Year	GP n (%)	KZN n (%)	MP n (%)	NW n (%)	WC n (%)	LP n (%)	FS n (%)	EC n (%)	NC n (%)	Totals n (%)
**2012**	618 (19)	201 (20)	30 (15)	15 (12)	2 (2)	13 (13)	0	0	0	**879 (100)**
**2013**	511 (15)	186 (18)	40 (20)	12 (10)	5 (5)	31 (32)	0	0	6 (27)	**791 (100)**
**2014**	521 (16)	196 (19)	32 (16)	16 (13)	1 (1)	15 (15)	5 (20)	6 (26)	3 (14)	**795 (100)**
**2015**	545 (16)	158 (15)	23 (11)	31 (25)	12 (12)	12 (12)	4 (16)	6 (26)	10 (45)	**795 (100)**
**2016**	519 (16)	138 (13)	39 (19)	19 (15)	41 (41)	15 (15)	11 (44)	6 (26)	3 (14)	**801 (100)**
**2017**	601 (18)	145 (14)	39 (19)	33 (26)	38 (38)	12 (12)	5 (20)	5 (22)	0	**791 (100)**
**Total**	**3315 (100)**	**1024 (100)**	**203 (100)**	**126 (100)**	**99 (100)**	**98 (100)**	**25 (100)**	**23 (100)**	**22 (100)**	**4935 (100)**

*EC* Eastern Cape; *FS* Free State; *GP* Gauteng; *KZN* KwaZulu Natal; *LP* Limpopo; *MP* Mpumalanga; *NW* North West; *NC* Northern Cape; *WC* Western Cape

## Discussion

In this paper, we report the number of tests positive for mumps in South Africa between 2012 to 2017. Most of these cases were reported by the private sector laboratories and occurred mostly in the 1–4 and 5–9 age groups. This age distribution is consistent with what has been reported in other countries during the pre-MuCV era, with most of the infections reported in children below 10 years of age [1]. The cumulative incidence of mumps in our setting was found to be lower than that reported in Western countries during the pre-vaccine era. This most likely reflects under-reporting of mumps since the disease is neither notifiable nor under surveillance. The low incidence could also mean that the reported cases represent patients with more severe presentation of the disease, such as mumps-associated meningitis or orchitis, in whom further investigations would have been conducted. The results could also indicate diagnostic practises in our setting, with mumps possibly only being diagnosed clinically if a patient presented with a typical presentation of parotid enlargement. This would suggest that mumps cases presenting with other complications of the disease could have possibly been undiagnosed and therefore not be accounted for in the analysed results. Also, had there been an outbreak of mumps during the study period, this may have been unidentified.

There have been recent reports on resurgence of mumps infections amongst adolescents and young adults in overcrowded and semi-closed settings such as communes, colleges and camps in developed countries [8–10, 14, 25]. In the United States (US), military recruits, a sub-population that has previously been associated with mumps outbreaks, were found not to be involved in the resurgence of mumps infections reported between 1998 and 2007 [8]. This was associated with the decision in 1991, to introduce the MMR vaccine amongst recruits irrespective of previous vaccination status. Although this finding could strengthen a case for booster doses in older age groups, particularly those at high risk such as college students, antibody titres have been found not to be durable, with titres returning to pre-MMR3 dose levels 1 year after vaccination in individuals between 18 and 24 years in a non-outbreak setting [9]. A booster dose of the mumps vaccination is currently recommended only in the setting of an outbreak [9, 10, 26–28]. Although the level of protective antibodies and correlates of protection against mumps infection are not well-defined, suggested causes of the resurgence of infections have included waning immunity over time due to a lack of a durable T-cell mediated response, as well as antigenic differences between vaccine and circulating mumps strains, [1, 4, 7–10, 26–31]. As such, the mismatch between vaccine and circulating mumps strains has also prompted the consideration of a polyvalent vaccine [1, 32].

In our study, most of the samples submitted for mumps testing were CSF and blood specimens. One study conducted in Gauteng Province in South Africa used CSF samples from patients who had clinical presentation of central nervous system disease (meningitis, encephalitis or other febrile illness with focal neurological signs) to determine the presence of mumps and to characterise the strains, if found [16]. The study found a low frequency of mumps-associated CNS disease [3/260 (1.2%)], and phylogenetic analysis of one detected strain showed that it was a Jeryl-Lynn or RIT4385 vaccine-like strain. A suggestion made by the authors was the establishing of a mumps surveillance programme in the country, which would also provide valuable mumps epidemiological data. At the time of submitting this paper, there was no established surveillance program for mumps in South Africa.

Our finding of a male predominance with regards to infections is similar to what has been reported in other studies [14, 33]. This has been associated with immunological differences between males and females, where females have been shown to have a stronger T-helper1 cell (Th1) immune response, as well as having persistent and higher antibody levels compared to males [31, 34]. Orchitis has been reported to be the most common complication of mumps infection, and this may also explain the higher proportion of males in this study [6]. Males have also been found to have an increased risk of complications that occur less commonly following mumps infections such as mumps-associated meningitis and encephalitis [6, 34].

The seasonal pattern of mumps infections differs by country, with this difference attributed to environmental, host and viral factors [35]. In our study, we found that the infections peaked in June and November. These months represent the beginning of winter and spring respectively in our setting [36]. A peak in infections in spring and winter has been reported in Jordan [35].

Although Western Cape was seemingly the most affected province with the highest average yearly incidence, the second highest number of recorded samples was from this province (second to Gauteng). Therefore, this province may have been over-represented in the analysis. The geographic distribution of the infections may also be due to the differential availability of laboratory services in the different provinces in the country.

Formulating recommendations for introducing a MuCV, one of the underutilized vaccines in the African region, in South Africa’s public health sector (through the EPI), is beyond the scope of this paper, and our study results are also not sufficient to inform such a policy [15]. As previously mentioned, before a MuCV can be introduced in a country, the baseline coverage of the measles-containing vaccine (MCV) should be > 80%. Based on the coverage data for the first and second doses of MCV (MCV1 and MCV2 respectively) in South Africa, the MCV1 coverage ranged from 68% in 2007 to 70% in 2018 and was > 80% only during the 2014–2016 period (84% in 2014, 86% in 2015 and 85% in 2016) [37]. The MCV2 vaccine coverage estimates (also from 2007 to 2018) showed a drastic decline, with estimates ranging from 49% in 2007 to 63% in 2016. Of note is that these quoted proportions are WHO and UNICEF estimates, and differ from the country’s official national and administrative estimates, all of which were > 80% for the MCV1 and between 70 and 95% for MCV2 between the 2007–2018 period. Further efforts in increasing the MCV uptake may therefore be required to meet the recommended baseline MCV vaccine coverage before considering the introduction of a MuCV [12, 38, 39]. Another important consideration regarding the introduction of a MuCV in South Africa’s public sector is the vaccine’s schedule compared to that of measles. The first dose of MuCV should be given between 12 and 18 months, and the second dose at the age of school entry (around 6 years of age), whereas, at the time that this paper was written, MCV was being given at 6 (first dose) and 12 months (second dose) according to the EPI schedule [13, 17]. Subsequent to the introduction of the MuCV, determining the effectiveness of the vaccine would be necessary. However, this could be challenged by the lack of knowledge regarding correlates of protection against mumps infection [26].

The main strength of our study is that we analysed data from both the public and private health sectors. However, our study had several limitations. Firstly, missing data could not be accounted for and information on risk factors was not available since the secondary data that was analysed did not include information about clinical and medical history. Secondly, 50% of mumps infections present non-specifically or with respiratory symptoms, while 20–40% of infections are reportedly asymptomatic or have mild symptoms [1, 27, 40]. These cases may not present at health facilities and would therefore not have been accounted for in the data that we reviewed. Also, data of cases of acute infection where the diagnosis was made clinically without laboratory confirmation would also not be included in our study. Since mumps was not a notifiable disease in South Africa at the time that this paper was written, case-based data that could have supplemented the laboratory-based data were also not available. The above-mentioned limitations may account for the small numbers of mumps test requests, particularly from the public health sector, where mumps infections are likely to be diagnosed clinically rather than by laboratory testing, due to consideration for resources. Thirdly, we were not able to comment on mumps-related complications in our setting because information on clinical presentation or medical history was not included in the analysed data. Fourthly, differential availability of laboratory services across the provinces may also have had an impact on the completeness of the analysed data. The estimates of acute infections presented may be an underestimation of the true burden of mumps disease and may explain why the cumulative incidence found in our study was lower than the cumulative incidence of ≥100 cases/100000 that has been reported in the pre-vaccine era in other settings.

## Conclusion

Our results showed that, in South Africa, mumps infections mostly affected children below 10 years of age, peaked during winter and spring and predominantly affected males. Fewer tests were performed in the public compared to the private sector, which may have contributed to under-reporting of infections. Since our study results were only based on laboratory test results, conducting further studies that include analysis of clinical data may provide further insight into disease burden in the country.

## Data Availability

The data used in the analysis are not publicly available. Public sector data were provided and approved for use by the data repository of the National Health Laboratory Service. Private sector data were provided and approved for use by the focal person (pathologists listed in the acknowledgement section) from the respective private sector laboratories [Dr Terry Marshall (Ampath Laboratories), Prof. Eftyxia Vardas (Lancet Laboratories), Dr. Inez Rossouw PathCare as well as Dr. Louis Marcus PathCare/Vermaak & Partners]. The datasets used and analysed during this current study are available from the corresponding author on reasonable request.

## Abbreviations

CNS: Central Nervous System
EC: Eastern Cape Province
EPI: Extended Immunization Programme
FS: Free State Province
GP: Gauteng Province
IgG: Immunoglobulin G
IgM: Immunoglobulin M
KZN: KwaZulu Natal Province
LP: Limpopo Province
MMR: Measles-mumps-rubella
MP: Mpumalanga Province
MuCV: mumps-containing vaccine
NA: nucleic acid
NC: Northern Cape Province
NHLS: National Health Laboratory Service
NPA: Nasopharyngeal Aspirate
NW: North West Province
US: United States of America
WC: Western Cape Province
WHO: World Health Organization
